# Hyperthermic intraperitoneal chemotherapy with cisplatin and mitomycin C for colorectal cancer peritoneal metastases: A systematic review of the literature

**DOI:** 10.1515/pp-2019-0006

**Published:** 2019-05-29

**Authors:** Amandine Pinto, Marc Pocard

**Affiliations:** Inserm U1275 - Carcinose Péritoine et Paris-Technologie, INSERM, Paris, France; U1275 - Carcinose Péritoine et Paris-Technologie, INSERM, Paris, France

**Keywords:** cisplatin, colorectal peritoneal metastasis, HIPEC, mitomycin C

## Abstract

**Background:**

The randomized trial PRODIGE 7 failed to show the benefit of oxaliplatin hyperthermic intraperitoneal chemotherapy (HIPEC) in colorectal peritoneal metastasis treatment (CR PM). This systematic review focuses on the association of cisplatin (CDDP) with mitomycin C (MMC) in HIPEC in CR PM.

**Content:**

Experimental studies demonstrated that hyperthermia, in addition to CDDP ± MMC treatment, gradually improved the cytotoxic effect by increasing early apoptosis, eATP interaction, intracellular CDDP concentration (by 20%) and p73 expression. Recent studies with highly selected patients reported unusual prolonged survival with a median overall survival (OS) of approximately 60 months, with a HIPEC combination of CDDP (25 mg/m^2^/L) plus MMC (3.3 mg/m^2^/L) at a temperature of 41.5–42.5 °C for 60–90 min. Major complications occurred in less than 30% of patients with limited hematological toxicity (less than 15%). In addition, in a phase 2 trial, an adjuvant HIPEC benefit was demonstrated in colorectal cancer patients with high risk for peritoneal failure (5-year OS: 81.3% vs. 70% for the HIPEC group vs. the control group, respectively, p=0.047). After a recurrence, an iterative procedure permitted similar recurrence-free disease (13 vs. 13.7 months) with an acceptable morbidity (18.7% of severe complications).

**Summary and outlook:**

The combination of CDDP and MMC seems to be an interesting protocol as an alternative to high-dose and short-term oxaliplatin.

## Introduction

Colorectal peritoneal metastasis (CR PM), with an occurrence rate of 40% [1, 2], is the second most common colorectal metastatic disease after hepatic metastases (HM) [3]. Historically, PM was considered a terminal disease. However, the development of combined treatment involving cytoreductive surgery (CRS) and hyperthermic intraperitoneal chemotherapy (HIPEC) permitted the consideration of peritoneal metastasis (PM) as a metastatic step eligible for locoregional treatment [4, 5, 6]. The fundamental goal of this additional chemotherapeutic treatment is to maximize the total drug concentration in the peritoneal tumor nodules with passive diffusion [7] while minimizing that delivered to the systemic circulation [7]. This treatment permits a median survival of more than 40 months [4, 5, 8, 9, 10]. However, the HIPEC protocol is not standardized. Several chemotherapeutic drugs have been used for CR PM treatment: oxaliplatin [11], mitomycin C (MMC) [11] or cisplatinium (CDDP). However, there is no consensus on the applied dose, temperature and duration, carrier solution, perfusate volume and technique (open vs. closed) [11, 12, 13]. Oxaliplatin-based HIPEC has been developed by French teams since 2000, whereas MMC is the most commonly used drug worldwide [4, 14, 15, 16]. The survival results seem to be comparable for these two drugs [17, 18, 19], but oxaliplatin was preferred by many teams because of the duration of the HIPEC protocol (30 min with oxaliplatin vs. 60 or 90 min with MMC), despite the increased risk of postoperative hemorrhage [20]. The number of published randomized trials on CRS and HIPEC is modest thus far, and this treatment will continue to face considerable methodological and practical challenges because of many HIPEC protocols, the choice of an adequate control group (CRS alone or combination chemotherapy?), the surgery in the experimental arm, and the long time period needed for the validity of the results [12, 13]. Recent results of the first phase III trial comparing CRS alone with CRS combined with HIPEC using oxaliplatin failed to demonstrate an overall survival advantage in the HIPEC arm, while the 60-day complication rate was significantly higher [21]. Ceelen proposed some potential explanations for the lack of benefit in the trial: oxaliplatin efficacy issues, adverse effects of intraperitoneal high dose glucose, and potential drawbacks of the use of hyperthermia [10]. Today, the PRODIGE 7 HIPEC protocol (oxaliplatin 30 min 360–460 mg m^2^ 43 °) is abandoned, and the protocol must be redefined: indication, chemotherapy, time of hyperthermia, etc. Recently, the effectiveness of CDDP has been validated in ovarian peritoneal metastasis in a phase III trial offering a HIPEC solution [22]. Is it possible that HIPEC with CDDP has a place in CR PM treatment? In the literature, we note that several teams associate CDDP with MMC in CR PM treatment [23, 24, 25, 26, 27]. Would this association optimize the effectiveness of MMC HIPEC? This systematic review focuses on the association of CDDP in combination with MMC for HIPEC in CR PM.

## Materials and methods

### Search strategy

This systematic review agrees with the guidelines outlined in the preferred reporting items for systematic reviews and meta-analyses statement (PRISMA). On PubMed, combinations of the following terms were used: “cisplatin,” “peritoneal metastasis,” “peritoneal carcinomatosis,” “colorectal cancer,” “experimental,” “hyperthermia,” “HIPEC” and “colon.” We first identified 105 articles with the combination of “peritoneal carcinomatosis” AND “colorectal cancer” AND “cisplatin” and 132 articles with the combination of “experimental” AND “hyperthermia” AND “cisplatin” words. To expand this search, we analyzed 63 articles involving “colorectal cancer” AND “hyperthermia” AND “cisplatin,” 50 articles involving “colon” AND “hyperthermia” AND “cisplatin”, and 37 articles with the combination of “colon” AND “HIPEC” AND “cisplatin”. An initial screening of the title and abstracts was performed.

**Figure 1:**
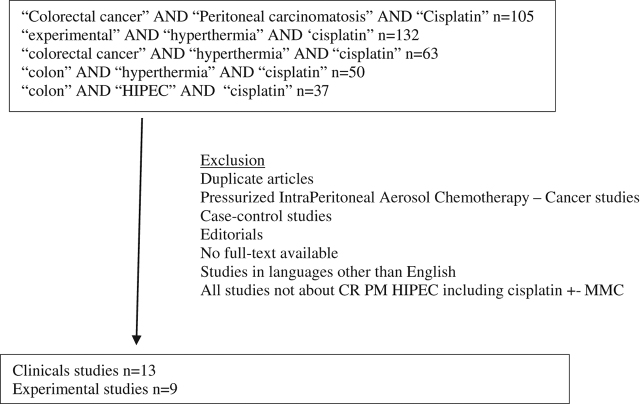
Flow chart.

### Eligibility and data extraction

Only studies including a surgical treatment of CR PM with HIPEC including cisplatin with/without MMC were selected. We first deleted duplicate articles and many articles about pressurized intraperitoneal aerosol chemotherapy – cancer (and no HIPEC). We separated experimental and clinical studies. The following studies were excluded: case-control studies, editorials, no full-text available, and studies in languages other than English. Finally, we analyzed 22 articles: 13 clinical and 9 preclinical studies (Figure 1).

For clinical studies, we extracted the study design, number of patients, peritoneal cancer index, follow-up, postoperative mortality and morbidity and survival.

## Results

### Experimental study

#### *In vitro* (Table 1)

Some experimental studies demonstrated that hyperthermia can affect cell membranes, the cytoskeleton, and the synthesis of macromolecules and can increase drug-induced DNA damage and inhibit the repair of drug-induced DNA damage [28]. CDDP appeared as a drug of choice associated with hyperthermia because hyperthermia provided pharmacokinetic advantages with higher local CDDP concentrations in tissues [29]. First, in gastric cancer cell lines, Tang demonstrated a synergistic effect on inhibiting proliferation and induction of cell death via the apoptotic pathway [30]. In 2018, Cesna validated this synergic effect on colon cancer cells [28]. Caco-2 cells (colon cancer cell line) were treated with CDDP. Hyperthermia gradually improved the cytotoxic effect and decreased the viability of cells by one-fourth from 43 °C to 45 °C. Furthermore, early apoptosis (20% compared to cells treated in normothermia) and an increase in the intracellular CDDP concentration (by 20%) were induced [28]. Some authors have investigated the role of adenosine triphosphate (ATP) in explaining the synergistic effect of chemotherapy and hyperthermia. ATP is basically undetectable in healthy tissues, whereas in the tumor interstitium, it was found in the hundreds of micromolar range [31]. Hyperthermia (40 °C) was noted to improve extracellular ATP-mediated cytotoxicity in MCA38 colon cancer cells [32]. With CDDP or MMC, hyperthermia and extracellular ATP together markedly potentiated cancer cell death [32]. The effects of hyperthermia on CDDP sensitivity were also proposed. Sotille [33] studied the effects of hyperthermia on CDDP sensitivity and determined whether MLH1 and MSH2 are associated with Hsp27 and Hsp72 in MMR-deficient(−)/–proficient(+) cells. We know that the MMR system (mismatch repair system) corrects mismatches and insertion/deletion loops generated during DNA replication, so the HCT116+ch2 (MMR−) and HCT116+ch3 (MMR+) cell lines were exposed to CDDP with or without previous hyperthermia (42 °C, 1 h). Whereas hyperthermia increased CDDP resistance in MMR-(1.42-fold), it potentiated CDDP sensitivity in MMR+  inducing cell cycle arrest and increasing p73 expression [33]. Sotille first suggested that p73 might participate in the cellular response to hyperthermia and CDDP in an MMR-dependent manner.

**Table 1:** Experimental studies about effect of hyperthermia and cisplatin ± mitomycin C; main results.

*IN VITRO*	Chemotherapeutic protocols	Aim and results
Cesna [28]	CDDP	Aim: to investigate the response to hyperthermia and chemotherapy
	Temperature: 37 °C to 45 °C	
		**Cytotoxicity: MTT assay**
		– 37 °C to 42 °C: no significant effect
		– 43 °C and 44 °C: viability dropped by 14 % and 20 %, respectively
		**Cell apoptosis: Annexin V-PE flow cytometry**
		CDDP: induce early apoptosis 1.5-fold
		CDDP+43 °C: induce early apoptosis as compared to cells treated in normothermia by 20 % (1 % of dead cells)
		**Intracellular concentration of cisplatin:**
		37 °C to 43 °C: the concentration was significantly increased by 20 %
Tang [30]	CDDP	Aim: to evaluate the impact of hyperthermia and hyperthermic chemotherapy on human gastric cancer cell lines and to explore the mechanisms of cell-killing effect
	Temperature: 37 °C or 43˚C	Synergistic effect of hyperthermia and CDDP on inhibiting proliferation in each cell line
		The cytotoxicity and proliferation inhibition of CDDP was dose-dependent / significant differences between normothermic chemotherapy and hyperthermic chemotherapy with a CDDP concentration range from 0 to 16 μg/mL.*p < 0.05; #p < 0.01.
		Hyperthermic chemotherapy induced cell death with two modes: apoptosis (more than 50 % of cell death occurred in early apoptosis) and necrosis
De Andrade Mello [32]	CDDP or MMC	Aim: to delineate the translatable strategy of hyperthermia to demonstrate impacts on P2X7 responsiveness to eATP
		Hyperthermia −> Increased membrane fluidity −> P2X7 hyperactivation −> potentiate pore opening and modulating downstream AKT/PRAS40/mTOR signaling events.
	Temperatures: 37 °C, 40 °C or 42 °C	Combination CDDP or MMC, hyperthermia and eATP −> potentiate cancer cell death
Sottile [33]	CDDPTemperature: 42 °C, 1 h	Aim: to study the effects of hyperthermia on CDDP sensitivity and to determine whether MLH1 and MSH2 associate with Hsp27 and Hsp72 in MMR-deficient(−)/–proficient(+) cells
		MMR- and MMR+cell lines were exposed to CDDP with or without previous hyperthermia
		**Clonogenic survival assay:**
		MMR- cells: hyperthermia increased CDDP resistance 1.42-fold (IC50=17.60 ± 2.10)
		MMR+cells: hyperthermia did not affect the number of colonies at concentrations lower than 10 mM, but it increased resistance at higher drug concentrations (IC50=11.50 ± 1.80)
		**Immunofluorescence**: to study Hsp27, Hsp72, MLH1 and MSH2 proteins after CDDP: Hsp27 and Hsp72 translocated to the nucleus and colocalization coefficients between these proteins with MLH1 and MSH2 increased in MMR+cells.
		**Western blotting and immunoprecipitation**: confirmed the interactions between HSPs and MMR proteins in control and treated cells
		**Cell cycle analysis**: hyperthermia pretreatment induced cell cycle arrest, increased p73 expression and potentiated CDDP sensitivity in MMR+cells.
Bhagwandin [34]	MMC, CDDP+DOX or CDDP	Aim: to evaluate the utility of *in vitro* drug sensitivity testing in patients with peritoneal surface malignancies undergoing CRS+HIPEC
	Alone	Data for 27 patients
		ChemoFx^®^ assay: results obtained and reported by Precision Therapeutics are grouped into three categories: (1) responsive; (2) intermediate-responsive; (3) nonresponsive
		*In vitro* chemosensitivity was noted in 17 patients (63 %).
		NO significant differences in OS and PFS for patients whose tumors displayed *in vitro* drug sensitivity versus those whose tumors did not (p=0.101 and p=0.403, respectively)
Makrin [35]	Male Wistar rats (n=96)	Aim: to examine the influence of chemotherapy and hyperthermia on the healing of colonic anastomosis
		Colonic anastomosis 2 cm above the ileo-cecal joint
		HIPEC protocol: closed / 40 °C /20 min
		**Bursting pressure of anastomoses** significantly lower than in controls (p < 0.01):
		Day 4 and 7:
		Surgery only: 54.8 and 170 mmHg
		HIPEC with saline: 38 and 188 mmHg
		HIPEC with MMC: 18 and 83 mmHg
		HIPEC with CDDP: 14.8 and 19 mmHg
		The difference decreased on day 10 and almost vanished on day 21
Aghayeva [36]	Wistar Albino rats (n=60)	Aim: to examine the influence of chemotherapy and hyperthermia on the healing of colonic anastomosis
		Sigmoid resection and end-to-end colorectal anastomosis
		HIPEC: open / 42 ° / 60 or 90 min
		**Bursting pressure of anastomosis**: no difference (p=0.81)
		Surgery only: 70 mmHg
		HIPEC with saline: 70 mmHg
		HIPEC with MMC: 60 mmHg
		HIPEC with CDDP: 80 mmHg
		HIPEC with OX: 70 mmHg
		HIPEC with DOX: 80 mmHg
Bevanda [37]	Mice	Aim: to investigate the effect of local chemoimmunotherapy and HIPEC in a mouse model of induced peritoneal metastasis
		IL-2 was IP injected at day 7 and 3 before implantation of tumour cells
		2 mL of saline heated to either 37 °C or 43 °C (hyperthermal treatment) and cytostatics followed tumoral IP injection
		Combined treatment with IL-2 and cytostatics (5-FU, CDDP or MMC) significantly increased the survival of mice:
		**ILS% – 37** **°C **= 29.88, 199.32, and 108.52, p=0.06, respectively
		**ILS% – 43** **°C **= 62.69, 260.50, and 178.05, p=0.01, respectively
Yun [38]	BALB/c mice (n=108)	Aim: to investigate the antitumor activity of a novel hydrogel drug delivery system through the combination of 5-FU loaded polymeric micelles and CDDP in biodegradable thermosensitive chitosan (CS) hydrogel.
		Colorectal peritoneal metastasis (CT26 IP) and IP treatment
		**The mean number and weight of tumor nodules:**
		CS hydrogel drug group (10.33 ± 2.66, 0.49 ± 0.11 g) were clearly decreased compared saline group (53.83 ± 9.99, 2.31 ± 0.38 g, p < 0.001), blank micelles–hydrogel group (52.67 ± 6.12, 2.26 ± 0.28 g, p < 0.001), 5-FU micelles group (22.5 ± 4.23, 0.99 ± 0.17 g, p < 0.001), CDDP loaded CS hydrogel group (23.33 ± 3.56, 0.98 ± 0.13 g, p < 0.001), FU+CDDP group (18.16 ± 3.06, 0.79 ± 0.13 g, p < 0.05)
		**The median survival:**
		CS hydrogel drug group (43 days) was remarkably longer than saline group group (25 days), blank micelles–hydrogel group (26 days), 5-FU micelles group (31 days), CDDP loaded CS hydrogel group (33 days), and FU+CDDP group (35 days)

MMC, mitomycin C; CDDP, cisplatin; OX, oxaliplatin; DOX, doxorubicine; IL-2, interleukin-2; 5-FU, 5-fluorouracil; IP, intra peritoneal; CS, biodegradable thermosensitive chitosan; ILS%, percentage of increased life span; CRS, cytoreductive surgery; HIPEC, hyperthermic intraperitoneal chemotherapy; OS, overall survival; DFS, disease free survival.

In view of these interesting *in vitro* results, Bhagwandin [34] performed a translational study and proposed to evaluate the utility of *in vitro* drug sensitivity testing in 27 patients with peritoneal surface malignancies (18.5% from colorectal origin, n=5) undergoing CRS plus HIPEC (chemotherapy agents included MMC or CDDP alone). Seventeen tumors (63%) displayed *in vitro* sensitivity to the agents used. However, there was no significant difference in survival for patients whose tumors displayed *in vitro* drug sensitivity vs. those whose tumors did not (p=0.101 and p=0.403, respectively). There was no correlation between *in vitro* drug sensitivity and the histopathology of the primary neoplasm (p=0.309). Today, it is not recommended to use these assays during the decision-making process in such patients because of the lack of clinical validation.

#### In vivo (Table 1)

The toxicity and effectiveness of the HIPEC procedure with MMC or CDDP were evaluated in preclinical studies.

Makrin showed that this procedure had a detrimental effect on the strength of colonic anastomosis, especially during the early postoperative period (until day 10) [35]. The bursting pressure of anastomoses in rats treated by HIPEC was significantly lower than in controls. On day 7, it was 170, 188, 83 and 19 mmHg in groups 1–4, respectively (p<0.01) (1: surgery only, 2: HIPEC with saline, 3: HIPEC with MMC, and 4: HIPEC with CDDP). This detrimental effect on anastomotic bursting pressure was not observed (p=0.81) in a recent rat model [36].

The effectiveness of these HIPEC procedures was evaluated in two experimental studies.

Bevanda suggested the synergistic effect of hyperthermia, chemotherapy and immunotherapy and that interleukin-2 (IL-2) significantly increased antitumor activity and the survival rate of mice with CR PM [37]. He compared the cytostatics 5-FU 150 mg/kg, CDDP 10 and MMC 5 mg/kg but did not test the CDDP-MMC association. Combined treatment with IL-2 and cytostatics (5-FU, CDDP or MMMC) significantly affected the development of peritoneal metastasis and increased the survival of mice: ILS% (*increased life span*) at 37 °C=29.88, 199.32 and 108.52, ILS% at 43 °C=62.69, 260.50 and 178.05, respectively). The most pronounced effect on survival was achieved by a combination of IL-2, CDDP and hyperthermia at 43 °C (ILS% at 37 °C=199.32 vs. 260.50 at 43 °C; p=0.01852, Kaplan-Meier analysis).

More recently, Yun proposed the development of a novel hydrogel drug delivery system through the combination of 5-fluorouracil (5-FU)-loaded polymeric micelles and CDDP in biodegradable thermosensitive chitosan (CS) hydrogels [38]. The results suggest that intraperitoneal administration of CS hydrogel drug can inhibit tumor growth in a mouse model of CR PM. It permitted prolonged survival time compared with other groups (p <0.05). The median survival in the CS hydrogel drug group (43 days) was longer than those in the NS (control) group (25 days), blank micelle–hydrogel group (26 days), 5-FU micelle group (31 days), CDDP loaded CS hydrogel group (33 days), and FU+CDDP group (35 days). Ki-67 immunohistochemical analysis revealed that tumors in the CS hydrogel drug group had lower cell proliferation in contrast to other groups (p < 0.001). Furthermore, hematoxylin-eosin staining of liver and lung tissue indicated that the CS hydrogel drug also had a certain inhibitory effect on colorectal cancer metastasis to the liver and lung.

Taken together, all experimental results suggest that CDDP and MMC are efficient drugs to control CR MP and that CDDP is more active in a hyperthermic situation.

### Clinical results

#### HIPEC protocol (Table 2)

**Table 2:** Hyperthermic intraperitoneal chemotherapy with cisplatin and mitomycin C for colorectal cancer peritoneal metastases: main results.

Author	Year	Patients, n	PCI (median)	Follow up (median)	Survival	Post-operative mortality / morbidity
Cavaliere [39]	2000	35 (n=11 CRPC)	16	17 mo	Median OS: 26 mo	Major complications: 37 %
					2 year OS: 55.2 mo – > 54.7 % for CRPC	
Cavaliere [40]	2000	40 (n=14 CRPC)	16	20 mo	Median OS: 30 mo	Major complications: 35 %
					2 year OS: 61.4 % – > 63.5 % for CRPC	
Cavaliere [41]	2011	146	< 11:33 %	19 mo	Median OS: 21 mo	Mortality: 3.4 %
			11–20:49 % > 20:18 %		2 year OS: 45 %	Major complications: 27.4 %
					2–3 year DFS: 33 % – 26 %	
Pilati [42]	2003	34		14.5 mo	Median OS: 18 m	Mortality: 0
					Median RFS: 13 mo	Morbidity: 36 %
					2 year OS – 2 year RFS: 31 % – 10 %	
Yonemura [43]	2013	142	≤ 10:53 % > 10:47 %		Median OS: 24 mo	Mortality: 0.7 %
		HIPEC: 87			5 year OS: 23.4 %	Major complications: 17.6 %
		No HIPEC: 55			Multivariate cox regression: CC score, histology, PCI ≤ 10	Morbidity: 42.9 %
Baratti [25]	2014	101	10	44.9 mo	Major morbidity: 5 year OS: 11.7 %	Mortality: 3 %
		Major morbidity: 24			No major morbidity: 5 year OS: 58.8 %	Major complications: 23.8 %
		No major morbidity: 77			Multivariate cox regression: major morbidity, PCI > 19, CC score	Reoperation: 10 %
Baratti [24]	2018	148	10	34.6 mo	PC+EPM:	Mortality: 3.4 %
		PC: 121	PC: 10		Median OS 19 mo	Major complications: 27.7 % −> 61 % cases presented
		PC + EPM: 27	PC + EPM: 8.5		Median RFS: 9.6 mo	PC+EPM
					5 year OS=16.5 %	Morbidity / systemic toxicity:
					PC alone:	– PC+EPM: 55.6 % / 27.8 %
					Median OS: 60.1 mo	– PC alone: 20.8 % / 3.8 %
					Median RFS: 13.8 mo	
					5 year OS=52 %	

Huang [26]	2014	60	21 ≤ 20:47 % > 20:53 %	29.9 mo	Median OS: 16.0	Mortality: 0 %
					5 year OS: 22 %	Major complications: 30.2 %
					1-2-3-5 year OS: 70.5 %-34.2 %-22 %-22 %	
					Multivariate cox regression: CC score, post-operative adjuvant chemotherapy	
Lin [27]	2016	31	16		2-5 year OS: 57 %-38 %	Mortality: 0 %
					Multivariate cox regression: PPCI score	Major complications: 0 %
						Morbidity: 21 %
Vaira [44]	2010	40	Group A (before 2002): 15,4		Group A: median OS 16.7 months	Mortality: 2.5 %
					Group B: median OS 24.6 months	Morbidity: 55 %
			Group B (2002–2008): 10,7			

OS, overall survival; DFS, disease free survival; RFS, recurrence free survival; Mo, months; CR PC, colorectal peritonealcarcinomatosis; HIPEC, hyperthermic intraperitoneal chemotherapy; PCI, peritoneal carcinomatosis index; PC, peritoneal carcinomatosis; EPD, extra peritoneal disease.

The HIPEC protocol with CDDP and MMC is not standardized. These two chemotherapeutic agents were mainly described by Italian teams. They were the first to publish this HIPEC protocol with oncological results. Cavaliere, in 2000 [39], published an open technique with CDDP (25 mg/m^2^/L) plus MMC (3.3 mg/m^2^/L) at a temperature of 41.5–42.5 °C for 90 min. The same year, he published a second study with the same protocol [40]. In 2011, he prospectively collected clinical data for 146 patients treated in five Italian hospitals for CR PM with CRS/HIPEC [41]. The choice of perfusion modality (open, semiclosed or closed) was left to the operator. However, the drugs perfused were the same as in the first studies, that is, CDDP 25 mg/m^2^/L of perfusate or CDDP 25 mg/m^2^/L plus MMC 3.3 mg/m^2^/L, and perfusion lasted 60–90 min at 41.5–43 °C. Baratti published three studies [23, 24, 25] evaluating the same HIPEC protocol with CDDP+MMC to treat CR PM. He proposed a closed-abdomen HIPEC protocol with the same drug concentrations for 60 min at a temperature of 42.5 °C. Pilati [42] published another Italian experience with this HIPEC protocol. Vaira in 2010 [44] proposed a HIPEC protocol according to the original semiclosed abdomen technique, with CDDP 100 mg/m^2^ plus MMC 16 mg/m^2^ at a temperature of 41.5 °C for 60 min.

Two Japanese teams described HIPEC with these two chemotherapeutic agents. Yonemura [43] used the same CDDP plus MMC HIPEC protocol as Cavaliere [39, 40] and Baratti [23, 24, 25]. Huang [26] used 120 mg of CDDP and 30 mg of MMC, each dissolved in 6 L of heated saline (drug concentrations: CDDP 20 mg/mL, MMC 5 mg/mL), for 90 min at a temperature of 43.0 ± 0.5 °C.

In 2016, Lin [27] published team results in Taiwan with 100 mg of CDDP plus 20 mg of MMC for 60 min at 42–43 °C.

#### Postoperative morbidity (Table 2)

Postoperative mortality after CRS/HIPEC CR PM treatment with CDDP ± MMC was described between 0% [42] and 3% [25], equivalent to the oxaliplatin HIPEC protocol [26]. Major complications occurred is less than 30% (27% in the more recent study) [23, 24, 25, 26, 27, 41, 42, 43, 44], mostly related to anastomotic leakage, intestinal fistula, abdominal abscess and pleura effusion, with approximately 10% reoperations. Hematological toxicity appeared in less than 15% [25, 42, 44] and hemorrhage between 1% [24] and 2% [26]. These results contrasted with oxaliplatin intraperitoneal toxicity. Hemoperitoneum (22.7%) and grade 3/4 thrombocytopenia (13.3%) were the most frequently reported toxicities with the oxaliplatin HIPEC protocol [45].

#### Long-term outcomes and recurrence (Table 2)

The median survival rates described for patients treated with HIPEC were very different, from 16 [26] to 60.1 months [22]. Five-year overall survival also appeared very different, between 22% [26] and 58.8% [25].

The peritoneal carcinomatosis index (PCI) could explain these survival differences. The majority of patients (53%) treated in Huang’s study [26] had an important PM (PCI > 20), with a median PCI at 21, whereas Baratti, in a recent study [24], showed survival of patients with a limited PM (median PCI=10). In a multivariate logistic regression analysis, Baratti confirmed that PCI > 19 (OR, 2.6; 95% CI, 1.1–6.0; p=0.02) was an independent predictive factor for major complications [25].

In addition, Baratti demonstrated that major complications independently affect long-term disease-specific survival (DSS) [25]. Five-year DSS was 14.3% (median, 18.5 months; 95% CI, 15.7–21.1) for patients who experienced major complications and 52.3% (median, 62.8; 95% CI, 23.9–101.7) for those who did not.

Extraperitoneal metastasis appeared as another pejorative survival criterion. In 2018, Baratti [24] compared the long-term outcomes between patients who had CRS/HIPEC for PM alone (n=121, 81.1%) and patients undergoing curative-intent treatments for extraperitoneal disease (EPD) (n=27, 18.2 %, 85 % had liver metastasis). The five-year OS was 52.0 % (median=60.1months; 95% CI, 44.9–93.7) for 121 patients treated for PM alone vs. 16.5% (median=19.0 months; 95% CI, 12.1–30.4) for 27 patients treated for EPD, p=0.019.

#### Prophylactic HIPEC

Two authors, namely, Virzi and Baratti, evaluated the HIPEC protocol with CDDP and MMC at the time of primary curative surgery in patients with colorectal cancer at high risk for metachronous peritoneal metastases [23, 46].

Virzi published [46] a prospective pilot study with 12 patients to assess the feasibility, safety and efficacy of this same HIPEC protocol combined with primary curative surgery in colorectal cancer at high risk for peritoneal metastases (minimal synchronous peritoneal involvement, synchronous ovarian metastases, primary tumor, either directly invading other organs or penetrating visceral peritoneum, *and* positive peritoneal washing cytological examination). The protocol was well tolerated and safe. Major morbidity occurred in 17% of cases and operative death in none. The 5-year progression-free and peritoneal progression-free survival rates were 74.1% and 90.9%, respectively. The 5-year overall survival was 83.3%, although one patient died shortly after 5 years.

Baratti [23] published a phase 2 study in 2016 that assessed adjuvant HIPEC in colorectal cancer patients at high risk for metachronous peritoneal metastasis (minimal synchronous peritoneal metastasis, synchronous ovarian metastases, primary tumor either penetrating the visceral peritoneum or directly invading other organs, age < =75 years, WHO performance score < = 2, no significant comorbidities, and signed informed consent). He included a number of patients included in the study by Virzi et al., as the latter was a preliminary report of the Italian experience with prophylactic HIPEC. A total of 22 patients without systemic metastases were prospectively enrolled in two centers to receive HIPEC simultaneously with curative surgery. A control group retrospectively included 44 matched (1:2) patients undergoing standard treatments. The Kaplan–Meier estimated 5-year overall survival (OS) was 81.3% in the HIPEC group vs. 70.0% in the control group (p=0.047). No operative death occurred, and severe morbidity rates were 18.2% in the HIPEC group and 25% in controls (p=0.75). In multivariate analysis, HIPEC correlated with a lower cumulative incidence of PM (hazard ratio [HR] 0.04, 95% CI 0.01–0.31; p=0.002), better OS (HR 0.25, 95% CI 0.07–0.89; p=0.039) and progression-free survival (HR 0.31, 95% CI 0.11–0.85; p=0.028). He concluded that adjuvant HIPEC may benefit these patients with a high risk of peritoneal failure.

#### What is the best treatment after a recurrence?

In some cases, after CR PM treatment by CRS/HIPEC, recurrences may be confined to the peritoneal cavity and are completely resectable. Vaira [47] evaluated the results of 16 patients presenting with isolated peritoneal recurrence who had undergone iterative CRS and HIPEC [47]. Only patients with PCI ≤ 16 and a progression-free interval of at least 12 months between the first HIPEC and the recurrence diagnosis and patients with completely resected disease received a second HIPEC. Colonic tumors perfused using 100 mg/m^2^ CDDP and 16 mg/m^2^ MMC at the first intervention were perfused at the second with 35 mg/m^2^ MMC. The median interval between the initial and second CRS with HIPEC was 19 months (range 12–111 months). The mean PCI was 8 vs. 16 for the first procedure. No patient died postoperatively, and the overall morbidity was 43.7%, with 18.7% severe complications. After a median follow-up of 20 months, RFS following repeated CRS and HIPEC was comparable (13 vs. 13.7 months) to that registered after the first procedure.

## Discussion

Residual microscopic metastases after cytoreductive surgery remain a therapeutic challenge. The adjuvant application of intraperitoneal chemotherapy has become the standard of care. However, the chemotherapeutic drug protocol is not standardized. In 2018, two randomized phase III studies evaluated combined therapy (CRS+HIPEC) vs. CRS alone for peritoneal metastases. PRODIGE 7 failed to show a difference in overall survival between patients treated with oxaliplatin HIPEC adjunction for CR PM. Overall survival was high in both groups but did not differ significantly (41.7 vs. 41.2 months in the HIPEC and control arm, respectively, HR 1.00 [95% CI: 0.73–1.37) [21]. In parallel, Van Driel [22] validated the effectiveness of HIPEC adjunction with CDDP in ovarian peritoneal metastasis. He showed the effectiveness of CDDP HIPEC on recurrence and overall survival [22]: 89% vs. 81% and 33.9 vs. 45.7 months for cytoreductive surgery alone or in association with HIPEC, respectively (p=0.003). These two major clinical trials have disturbed the expert community [48]. The PRODIGE 7 results were the object of intensive discussions among HIPEC surgeons during the last meeting of the Peritoneal Surface Oncology Group International (PSOGI) in Paris on 9–11 September 2018 [48]. Ceelen [10] and other experts [48] proposed some potential explanations for the lack of benefit, including oxaliplatin efficacy issues, adverse effects of intraperitoneal high dose glucose, and potential drawbacks of the use of hyperthermia. The HIPEC protocol for the treatment of CR PM must be redefined: indication, chemotherapy and time of hyperthermia. We were interested in CDDP’s place in this indication. Today, we have no comparative data showing the superiority of MMC alone or associated with CDDP over other HIPEC regimens or a survival advantage of CRS-HIPEC with MMC over modern palliative systemic chemotherapy. Few experimental studies evaluated this protocol in CR PM, but they suggested that CDDP and MMC were efficient drugs to control CR MP and that CDDP was more active in a hyperthermic situation. The most frequently described protocol consists of the combination of CDDP (25 mg/m^2^/L) plus MMC (3.3 mg/m^2^/L) at a temperature of 41.5–42.5 °C for 60–90 min. The survival results described for patients treated with HIPEC combined with CDDP and MMC were very different. We first explain these differences by the peritoneal carcinomatosis index (PCI), which is a very different function of cohorts. However, PCI has been demonstrated by many multivariate analyses to be the most important adverse prognostic factor after CRS-HIPEC [24, 25, 27, 43, 49]. It is important to note that patients treated for PM alone had a very important OS survival (60 months), equivalent or higher than that of patients receiving oxaliplatin IP [10, 19, 50]. Concerning HIPEC morbidity with CDDP, Van Driel [22] did not note an increasing risk of postoperative complications (major morbidity: 25% and 27% for surgery alone vs. the surgery-plus-HIPEC group, p=0.76). In the CR PM literature, major complications occurred in less than 30% (27% in the more recent study). Taken together, all results indicated that the drug concentration, carrier solution, level of hyperthermia or duration is possibly related to the postoperative high complication rate level. For example, 5% dextrose, an oxaliplatin carrier solution, causes metabolic and electrolyte shifts (hyperglycemia and hyponatremia), which may exacerbate surgical morbidity [10]. No clinical studies comparing normothermic with hyperthermic chemoperfusion permit validation of the adverse effects of hyperthermia. However, it is admitted that toxicities within the peritoneal cavity, such as small bowel fistula and anastomotic leakage, undoubtedly increase as the area under the curve for drug dose increases [6]. Regarding the effectiveness and safety, this Italian HIPEC protocol with a combination of CDDP (25 mg/m^2^/L) plus MMC (3.3 mg/m^2^/L) at a temperature of 41.5–42.5 °C for 60–90 min could be the rational base to support the design of future randomized trials vs. cytoreductive surgery alone in the treatment of colorectal PM. This protocol represents a potential alternative for now. We expect, in the next 10 years, new drug delivery systems, immunotherapy combined with chemotherapeutic, and development of nanovectorization, a new therapeutic area, to improve actual survival and decrease morbidity [51].

Today, the CDDP+MMC-based HIPEC is the only protocol to demonstrate an adjuvant HIPEC benefit in colorectal cancer patients at high risk for peritoneal failure (5-year overall survival: 81.3% vs. 70% for the HIPEC group vs. the control group, respectively, p=0.047) [23]. During 2006–2012, a total of 22 patients without systemic metastases were prospectively enrolled to receive HIPEC simultaneously with curative surgery. A control group retrospectively included 44 matched (1:2) patients undergoing standard treatments and no HIPEC during the same period. PROPHYLOCHIP, a phase III randomized trial, evaluated surveillance vs. a systematic surgical look with oxaliplatin HIPEC at the end of adjuvant chemotherapy for colorectal cancers operated with a high risk of peritoneal metastasis (perforated tumor, ovarian metastasis, minimal peritoneal disease resected at the same time as the primitive). The results were presented at the 2018 ASCO (American Society of Clinical Oncology) meeting (Goéré, A3531). The Tris trial randomized 150 patients between surveillance and second surgery. Disease-free survival and overall survival at 3 years were not different: 44% vs. 51% and 79% vs. 80%, NS, (second look vs. surveillance, respectively).

The combination of CDDP and MMC might be a valid HIPEC protocol in CR PM. Recent studies evaluating this protocol demonstrated prolonged survival (overall survival approximately 60 months) with limited toxicity. Major complications occurred in less than 30% of cases with few hematological toxicities (less than 15%). This protocol is the only one to demonstrate an adjuvant HIPEC benefit for CRC patients at high risk for peritoneal failure (5-year overall survival 81.3% vs. 70% for the HIPEC group vs. the control group, respectively [p=0.047]). After a recurrence, an iterative procedure is possible with similar recurrence-free disease (13 vs. 13.7 months) and acceptable morbidity (18.7 % severe complications). Randomized trials are now needed to confirm this hypothesis.
